# IL-22Ra1 is induced during influenza infection by direct and indirect TLR3 induction of STAT1

**DOI:** 10.1186/s12931-019-1153-4

**Published:** 2019-08-15

**Authors:** K. D. Hebert, N. Mclaughlin, Zhe Zhang, A. Cipriani, J. F. Alcorn, D. A. Pociask

**Affiliations:** 1 Department of Medicine, Pulmonary Diseases, Critical Care & Environmental Medicine, Tulane University School of Medicine, 1430 Tulane Ave. Mail code sl279, New Orleans, LA 70112 USA; 2 Department of Pediatrics, Children’s Hospital of Pittsburgh of UPMC, One Children’s Hospital Drive, 4401 Penn Ave, Pittsburgh, PA 15224 USA

**Keywords:** Influenza, IL-22Ra1, IL-22, STAT1, TLR3, IFNβ

## Abstract

**Background:**

Influenza attacks the epithelium of the lung, causing cell death and disruption of the epithelial barrier leading to fluid buildup in the lung and impairment of gas exchange. Limited treatment options for severe influenza pneumonia prioritize the need for the discovery of effective therapies. IL-22 is a cytokine that promotes tissue integrity and has strong promise as a treatment option. While research has been focused on the cytokine itself, there is limited understanding of the regulation of the IL-22 receptor (IL-22Ra1) at the epithelial surface during infection.

**Methods:**

IL-22Ra1 levels were measured by qRT-PCR, western blot and immunofluorescence following H1N1 influenza infection (A/PR/8/34 H1N1) or synthetic TLR3 mimetic, Poly (I:C). Regulation of the receptor was determined using STAT inhibitors (STAT1, STAT3 and PanSTAT inhibitors), TLR3 inhibition, and neutralization of interferon alpha receptor 2 (IFNAR2). Significance was determined by a *p*-value of greater than 0.05. Significance between two groups was measured using unpaired t-test and significance between more than two groups was measured using one-way ANOVA with Tukey Multiple Comparison Test.

**Results:**

Here we show both in vivo and in vitro that IL-22Ra1 was induced as early as 24 h after influenza (H1N1 PR8) infection. This induction was triggered by toll-like receptor 3 (TLR3) as a TLR3 mimetic [Poly (I:C)] also induced IL-22Ra1 and inhibition of endosomal formation required for TLR3 function inhibited this process. This upregulation was dependent upon IFNβ signaling through STAT1. Importantly, induction of IL-22Ra1 significantly increased IL-22 signaling as evidenced by pSTAT3 levels following IL-22 treatment.

**Conclusion:**

Collectively, these data suggest epithelial cells may optimize the beneficial effects of IL-22 through the induction of the IL-22 receptor during viral infection in the lung.

## Background

The lung acts as a filter, removing harmful particulate and infectious material to allow efficient gas exchange at the alveolar surface. The pulmonary lining consists of various specialized epithelial cell populations that serve as vital barrier between the outside world and the blood stream. Equally important, epithelial cells also contribute to the innate host defense through the production of mucus, antimicrobial molecules and mucociliary clearance. While these defenses are often sufficient for preventing injury and infection, specialized pathogens such as influenza A virus (IAV) have developed ways to infect the epithelial cells of the lung, leading to epithelial stress [1], loss of barrier function [2] and cell death [3].

Influenza A virus (IAV) is a member of the Orthomyxoviridae family. It is an RNA virus that is highly adapted to infect the pulmonary epithelial cells through binding to sialic acid residues. While IAV commonly infects the upper airways, variants such as H3N2 and H5N1 can infect the lower airways and alveolar epithelium [4, 5]. Once the virus infects a cell it proceeds to hijack the cellular machinery for viral proliferation. To combat this, host cells have developed a class of receptors known as Toll-like receptors (TLRs) to identify pathogen associated molecular patterns (PAMPs). In the case of influenza, it is the TLR3 that acts as an early warning system for the infected epithelial cells [6]. TLR3 is an intracellular receptor that is expressed on the endosomal membrane. Upon infection, TLR3 recognizes double stranded RNA (dsRNA), leading to signaling pathways that drive the production of Type I interferons (IFNs) [7]. Release of IFNα and IFNβ act upon neighboring epithelial cells to alert them to the infection, instigate intracellular anti-viral pathways as well as initiate the innate and adaptive immune responses [8].

Severe influenza infection can lead to damage of the pulmonary epithelium. To reduce this injury, a number of growth factors and cytokines, such as interleukin 22 (IL-22) are released to help prevent epithelial loss and promote repair. IL-22 is a member of the IL-10 family of cytokines. One of the identifying features of this family is the receptor complexes used. In the case of the IL-22 receptor, it is a heterodimer of the IL-10Rb and IL-22Ra1 [9]. In the lung, IL-10Rb is ubiquitously expressed on the epithelial cells, while IL-22Ra1 is found predominantly on airway cells under naïve circumstances [10, 11]. During influenza infection, the IL-22Ra1 is produced by type II cells in areas of parenchymal injury and inflammation [10]. Moreover, mice lacking IL-22 (*Il22*^−/−^) demonstrate reduced airway and parenchymal repair and increased scarring [10]. These data demonstrate that IL-22 is vital for epithelial repair during infection. Moreover, upregulation of IL-22Ra1 may be an important mechanism to promote epithelial repair by allowing IL-22 to signal to cells that are undergoing infection, stress or injury.

Given the potential of IL-22 to restore the pulmonary barrier and restore lung function after infection, we believe it is important to understand the cells upon which IL-22 will act and mechanisms in which the IL-22Ra1 produced. Here we demonstrate both in vivo and in vitro that IL-22Ra1 is induced rapidly after influenza infection in a TLR3 dependent manner. Receptor induction requires type I interferon signaling through STAT1. Induction of IL-22Ra1 is important as it allows cells to be more sensitive to IL-22 and leads to greater activation of IL-22 signaling through STAT3.

## Methods

### Cell culture

A549 cells and c10 cells were cultivated in Dulbecco’s modified Eagles medium (DMEM) with 10% fetal bovine serum (FBS) and 1% penicillin/streptomycin. BEAS-2B and primary normal human bronchial epithelial (NHBE) cells were cultivated in Bronchial Epithelial Cell Basal Medium (BEBM) with BEGM SingleQuots (Lonza, Allendale NJ) and 1% penicillin/streptomycin. For primary cell lines, all plates were coated with .01 mg/ml human fibronectin (Corning, Oneonto NY), 0.03 mg/ml bovine collagen type I (Advanced Biomatrix, Carlsbad, CA), and 0.01 mg/ml bovine serum albumin (Fisher Bioreagents, Waltham MA) in serum free BEBM. For maintenance of all stock cultures, cells were grown to ~ 70% confluence then dissociated using 0.25% Trypsin-EDTA (Gibco, Waltham MA). For A549 and c10 cells, they were resuspended in DMEM with 10% FBS (Gibco, Waltham MA) and 1% Pen Strep (Gibco, Waltham MA) then plated. For primary cell lines, after dissociation cells were transferred to a 50 ml conical then spun down at 500 g for 5 min. Trypsin was then aspirated and fresh BEBM growth media added before plating cells.

### Mice

6–8 week old WT (C57Bl/6) and STAT1^−/−^ (a kind gift from Dr. John Alcorn) male mice were used for all experiments and housed in pathogen free conditions in accordance with Tulane University’s Institutional Animal Care and Use Committee.

### Oropharyngeal administration of influenza (a/PR/8/38 H1N1) or poly (I:C)

All treatments were performed via oropharyngeal aspiration while mice were under isofluorane anesthesia. Poly (I:C) (Invivogen, San Diego, CA) was administered (50 μg/ml) in 100 μl of sterile water. A/PR/8/34 H1N1 (PR8) was administered at 100PFU in 50ul of sterile PBS. This is a sublethal dose that has been well characterized by our lab [10, 12]. Typically, mice begin losing weight 4 days after infection and recover full weight within 14 days. Notably, this model induces long term epithelial changes that can be found as late as 60 days after infection [12].

### Bronchial brushings

Mice were sacrificed by CO_2_ inhalation and cervical dislocation was performed. The rib cage was removed to visualize the lungs. The trachea was exposed and an incision was made to remove the viscera and muscle around it. Carefully, a small incision was made into the trachea and abraded PE-10 polyethylene tubing (BD Biosciences, San Jose, CA) was then used to brush inside each branch of the right and left side of the upper airways. One piece of tubing was used to brush each side respectively. Tubing from each bronchial brushing was transferred into 200 μl Trizol then flash frozen until RNA isolation as described in RT-qPCR section. Presence of upper airway cells were confirmed via RT-qPCR for *Scgb1a1*.

### Poly (I:C) and Pr8 treatment of cells

Cells were seeded in 6 well plates at 3.0 × 10^5^ cell/well and allowed to grow near 70% confluence in their respective growth media (DMEM or BEBM). Once 70% confluence was reached A549 and c10 cells were serum starved in DMEM with 1% FBS. After 24 h cells were then treated with Poly (I:C) (50 μg/ml) or Pr8 (MOI: 50) and harvested in Trizol (Life Technologies, Carlsbad, CA) for RNA extraction or fixed in 4% PFA for immunofluorescence analysis.

### STAT inhibition assays

STAT inhibitors (Fludarabine, Stattic, and Nifuroxazide) were purchased from Selleck (Houston, TX). For STAT3 and PanSTAT inhibitor assays A549 cells were pretreated for 3 h with 1 μM Stattic or Nifuroxazide respectively. For STAT1 inhibitor assays A549 cells were pretreated for 24 h with 1 μM Fludarabine. After pretreatment cells were then treated with 30 units of IFNβ (R&D systems, Minneapolis, MN) for 6 h then collected in Trizol (Life Technologies, Carlsbad, CA). For STAT inhibitor + Poly (I:C) (50 μg/ml) experiments, cells were harvested in Trizol after 12 h. Toxicity of each STAT inhibitor was determined by MTT assay on A549 cells using varying concentrations of each inhibitor in a 96 well plate. MTT reagents (Trevigen, Gaithersburg, MD) were added according to manufacturer’s protocol and absorbance wavelengths were read at 690 nm and 540 nm on an Epoch plate reader (Biotek, Winooski, VT). Viability % was determined by the following equation: [(untreated control absorbance ─ treatment group absorbance) ÷ untreated control absorbance] × 100.

### TLR3 inhibitor and αIFNAR experiments

TLR3 inhibitor (Chloroquine) was purchased from Invivogen (San Diego, CA). αIFNAR2 (MMHAR-2) was purchased from EMD Millipore (Burlington, MA). For both experiments A549 cells were pretreated for 30 min with Chloroquine at 15 μg/ml or αIFNAR2 at 5 μg/ml respectively. Cells were then treated with Poly (I:C) (50 μg/ml) and collected in Trizol after 12 h.

### pSTAT3(Y705) assay

BEAS-2B and NHBE were seeded in 6 well plates at 3.0 × 10^5^ cell/well and allowed to grow near 70% confluence in their growth media (BEBM). Cells were then treated with IFNβ (30 U/ml) for 24 h. After 24 h cells were treated with IL-22 (20 ng/ml) for 15 min then isolated in RIPA buffer (Thermo Scientific, Grand Island, NY) with Protease and Phosphatase inhibitors (Thermo Scientific, Grand Island, NY). Cells were scraped then sonicated at 15% in their respective tubes for one 15 s burst. Samples were spun down at 14,000 g for 15 min and supernatant transferred to new respective tubes. Protein was quantified and normalized via Bradford assay and loaded onto 4–12% NuPAGE Bis-Tris gel at 15μg of protein per sample. Transfers were done on iBlot (Invitrogen, Carlsbad, CA). pSTAT3 (Y705) antibody (Cell Signaling Technologies, Danvers, MA) was used to detect classically activated phosphorylated STAT3.

### Immunofluorescence

A549 cells were infected on coverslips for 24 h at MOI 50. After 24 h cells were then fixed in 4% paraformaldehyde for 10 min. After washing with PBS cells were permeabilized with 0.2% Triton X-100. Cells were then blocked in 5% normal goat serum and stained for IL-22Ra1 (Invitrogen, Carlsbad, CA) at 10 μg/ml for 2 h. Goat anti-rabbit 488 (Invitrogen, Carlsbad, CA) was then used as a secondary and counterstained with DAPI. EVOS FL Auto Imaging System was used for analysis. Quantification was done using ImageJ. Total mean fluorescent intensity of IL-22Ra1 was divided by the total number of cells per field (6 samples per condition, 20 pictures each).

### RT-qPCR

RNA isolation was performed on cells and bronchial brushings using Trizol method (Life Technologies, Carlsbad, CA). Briefly, 200 μl of chloroform and 200ul of sterile PBS was added to each sample and shaken vigorously for 30 s. Samples were incubated for 10 min then spun down at 12,000 g for 15 min at 4 °C. The aqueous phase was then placed into 500 μl isopropanol, mixed lightly and incubated for 5 min. Samples were then spun down at 12,000 g for 10 min at 4 °C. Supernatant was decanted then 1 ml of 75% ethanol was added to each sample and spun down at 7600 g for 5 min. This step was repeated twice then RNA pellet was allowed to air dry before adding 30 μl of nuclease free water. RNA was quantified by Nanodrop and quality was determined by a 260/280 verification of ~ 2. One microgram of RNA was reverse transcribed using iScript (Bio-Rad, Hercules, California) and verified by RT-PCR amplification of the GAPDH housekeeping gene.

TaqMan Gene Expression primers (Applied Biosystems) were used to determine levels of: GAPDH (Hs02758991_g1), IL-22Ra1 (Hs00222035_m1), and IFNβ1 (Hs01077958_s1).

### Statistical analysis

All data are represented as the mean ± SEM. Significance was determined using either an unpaired two-tailed t-test when comparing two groups or a one-way ANOVA with Tukey adjustment when comparing multiple groups. All statistics were calculated using GraphPad Prism 6.

## Results

### *IL-22Ra1* induction following H1N1 infection is TLR3 mediated

**Fig. 1:**
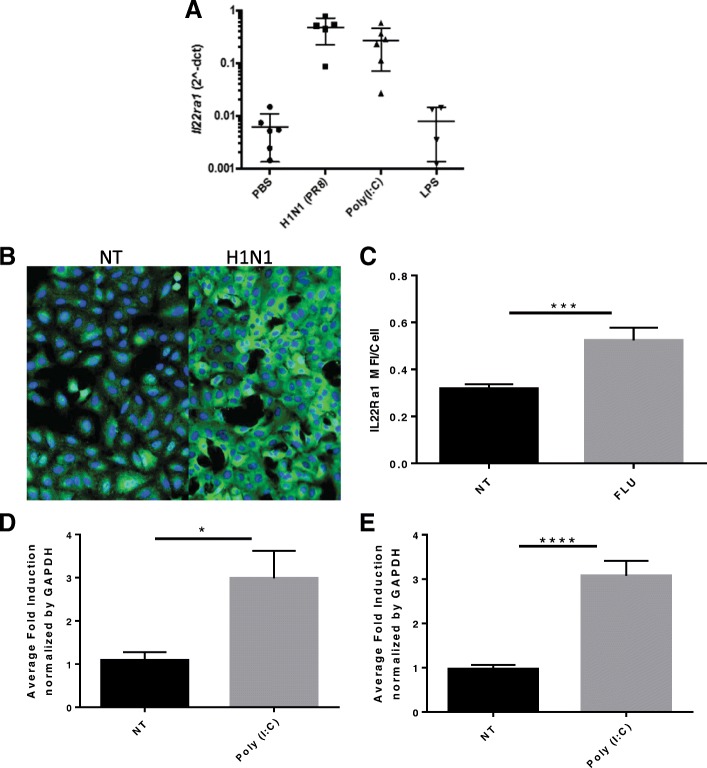
TLR3 mediated induction of *IL-22Ra1* by Poly (I:C) and H1N1 in vitro and in vivo. **a** Mice were treated oropharyngeally with Poly (I:C) (50μg/ml), H1N1 (100 pfu) or LPS (2 μg/ml) for 24 h respectively then bronchial brushings were performed. RNA was then isolated for RT-qPCR analysis of *Il-22ra1*. Both Poly (I:C) and H1N1 significantly induced *Il-22ra1*. (one way ANOVA *p* < 0.0001). Induction was also confirmed by immunofluorescence of IL-22Ra1 in H1N1 infected (MOI 50) A549 cells. **b** Untreated control vs. H1N1 24 h.p.i (both at 20x magnification). **c** Quantification of immunofluorescent IL-22Ra1 induction [t-test, *p* = 0.0005,*n* = 6 (20 pictures per condition)]. Representative of two experiments. Cells were treated with Poly (I:C) (50μg/ml) or H1N1 (MOI 50) respectively then RNA was then isolated for RT-qPCR analysis of *IL-22Ra1* in **d**.) A549s (t-test *p* = 0.0167, *n* = 6) and **e**.) BEAS-2Bs, a primary airway cell line (t-test *p* < 0.0001,*n* = 9)

**Fig. 2:**
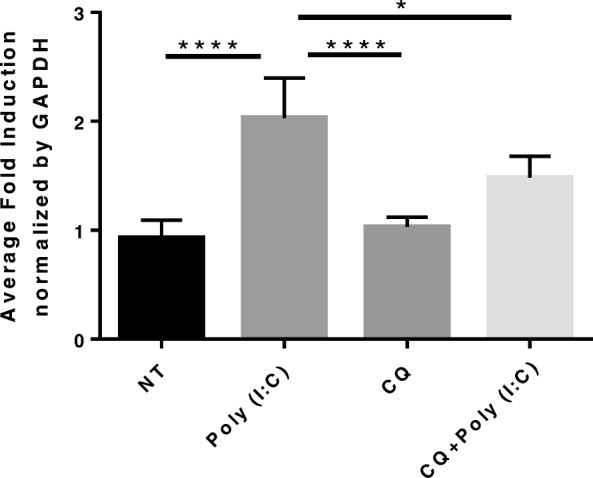
*IL-22Ra1* induction is TLR3 mediated. Cells were pretreated with Chloroquine (CQ) (15μg/ml) for 30 min that treated with Poly (I:C) (50μg/ml). Chloroquine pretreatment prevented TLR3 induction of *IL-22Ra1* in both A549s after 12 h (data not shown) and NHBEs after 24 h (one-way ANOVA, * = 0.02, **** < 0.0001, *n* = 4). Representative of three experiments

We have reported that IL-22Ra1 is significantly induced in both the airways and lung parenchyma following H1N1 infection [10]. However, the mechanisms that drive this phenomenon are still unclear. TLR3 recognition of influenza is part of the epithelial cells immediate response to infection. To determine if IL-22Ra1 is induced through TLR3, C57Bl/6 mice were infected with influenza (H1N1 PR8) or treated with a synthetic dsRNA TLR3 agonist Poly(I:C). A significant increase in transcript levels of *Il-22ra1* was measured within 24 h from RNA collected from bronchial brushings in mice both infected with Pr8 and treated with Poly(I:C). Interestingly this induction seems specific to TLR3 as endotoxin (LPS), which is recognized by TLR4, did not induce an increase in the receptor by RT-qPCR (one-way ANOVA, *p* < 0.0001) (Fig. 1a). This was confirmed in vitro as immunofluorescence for IL-22Ra1 showed protein induction 24 h after H1N1 infection in A549 cells (MOI 50) (Fig. 1b and c). Further, Poly (I:C) significantly induced *IL-22Ra1* gene expression as early as 12 h in A549s (two tailed t-test, *p* = 0.0167) (Fig. 1d) and after 24 h in BEAS-2Bs (two tailed t-test *p* < 0.0001) (Fig. 1e) respectively. Moreover, this induction was found to be specific to TLR3 as inhibition of TLR3 by Chloroquine, an endosomal acidification inhibitor, prevented induction of *IL-22Ra1* by Poly (I:C) in primary NHBE cells (one-way ANOVA, *p* = 0.02) (Fig. 2).

### TLR3 mediated upregulation of IFNβ drives rapid induction of *IL-22Ra1*

**Fig. 3:**
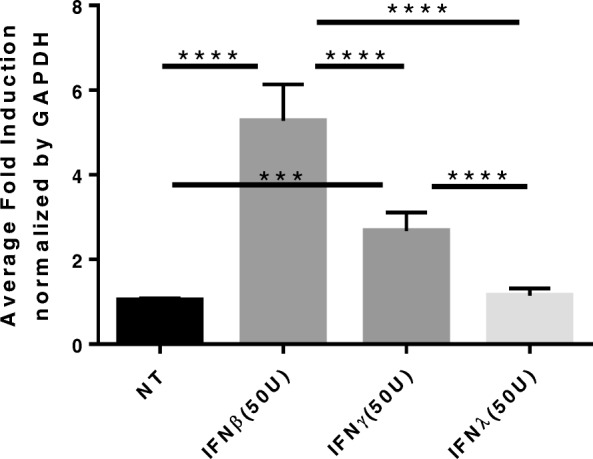
*IL-22Ra1* induction by Type I and Type II interferons but not Type III. A549s were treated with 50 units/ml of IFNβ, IFNγ, and IFNλ respectively. Cells were collected in trizol after 6 h and RNA isolated for RT-qPCR analysis. IFNβ treatment lead to significant induction of *IL-22Ra1* when compared to all other groups (one-way ANOVA, *p* < 0.0001). IFNγ treatment led to lower albeit significant induction of *IL-22Ra1* than IFNβ (one-way ANOVA, *p* = 0.0002). IFNλ treatment had no effect on expression of *IL-22Ra1*. Representative of two experiments

**Fig. 4:**
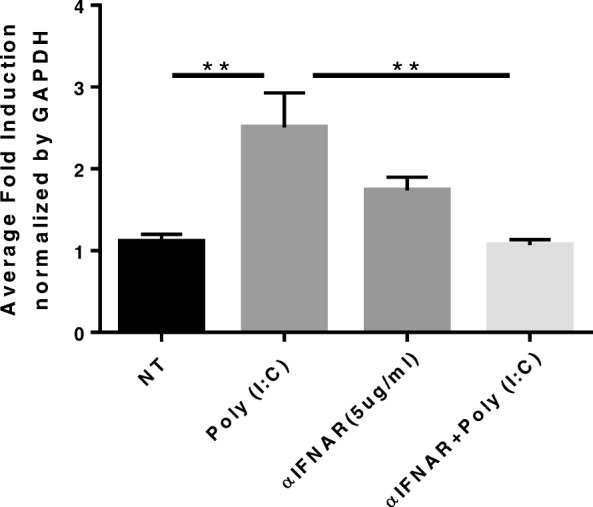
Type I interferon signaling is responsible for *IL-22Ra1* induction following H1N1 infection. A549s were pretreated with 5 μg/ml of anti-IFNα receptor 2 antibody, αIFNAR2, for 30 min. Cells were then treated with Poly (I:C) (50 μg/ml) for 12 h. Cell were then collected in trizol and RNA isolated for RT-qPCR analysis of IL-22Ra1. αIFNAR2 inhibited induction of IL-22Ra1 confirming that Type I interferon signaling was responsible for induction of IL-22Ra1 following H1N1 infection (one-way ANOVA, *p* = 0.0055). Representative of two experiments

Given that TLR3 activation leads to release of Type I interferons, we tested whether IFNβ treatment would lead to direct induction of *IL-22Ra1*. Type II interferon (IFNγ) and Type III interferon (IFNλ) were also administered to determine if *IL-22Ra1* induction was Type I interferon (IFNβ) specific. As predicted, Type I interferon treatment significantly induced *IL-22Ra1* expression when compared to all other groups (one-way ANOVA, *p* < 0.0001) (Fig. 3). These data led us to hypothesize that TLR3 induces IL-22Ra1 in an interferon dependent manner. To test this, we administered an anti-IFNaR2 antibody, which inhibits the type I interferon receptor 2 (IFNaR2). Results showed that upon inhibition, there was a loss of *IL-22Ra1* induction by poly(I:C) (one-way ANOVA, *p* = 0.0055) (Fig. 4).

**Fig. 5:**
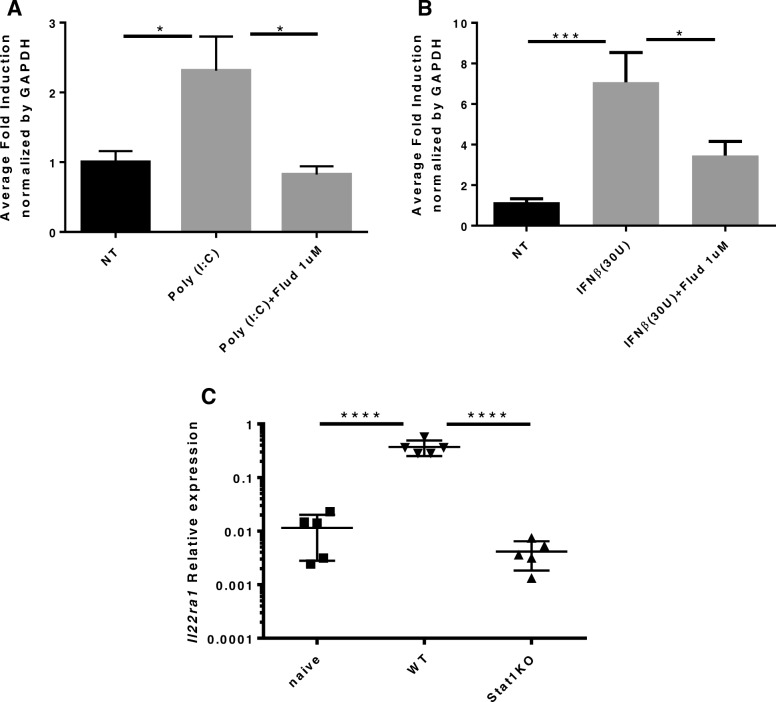
STAT1 is required for *IL-22Ra1* induction in vivo and in vitro**.** A549s were pretreated with 1 μM of STAT1 inhibitor, Fludarabine, for 24 h. Cells were then treated with Poly I:C (50 μg/ml) for 12 h or IFNβ (30 U/ml) for 6 h respectively. Cell were then collected in trizol and RNA isolated for RT-qPCR analysis of *IL-22Ra1*. Fludarabine inhibited induction of *IL-22Ra1* by both **a** Poly (I:C) (one-way ANOVA, *p* = 0.0372, *n* = 4) and **b** IFNβ (one-way ANOVA, * ≤ 0.05,*** = 0.001, *n* = 8). **c** C57Bl/6 and STAT1−/− mice were given Poly (I:C) (50 μg/ml) oropharyngeally and bronchial brushings were performed 24 h later. STAT1−/− failed to induce *Il-22ra1* (one-way ANOVA **** < 0.0001). Representative of two experiments

**Fig. 6:**
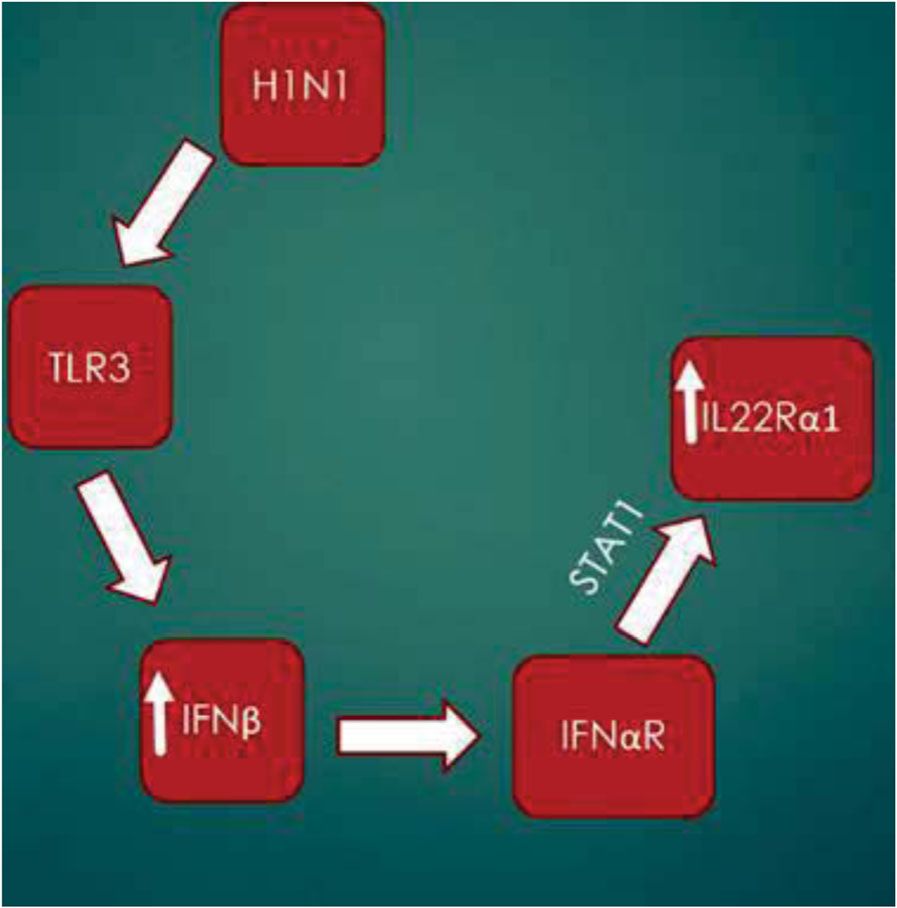
Proposed mechanism of H1N1 induction of *IL-22Ra1*. H1N1 activates TLR3 via its dsRNA. TLR3 activation then increases levels of IFNβ. IFNβ then acts through its receptors, IFNαR1 and IFNαR2, and phosphorylates STAT1. pSTAT1 then dimerizes (likely with STAT2) and activates promoter upstream of *IL-22Ra1*

**Fig. 7:**
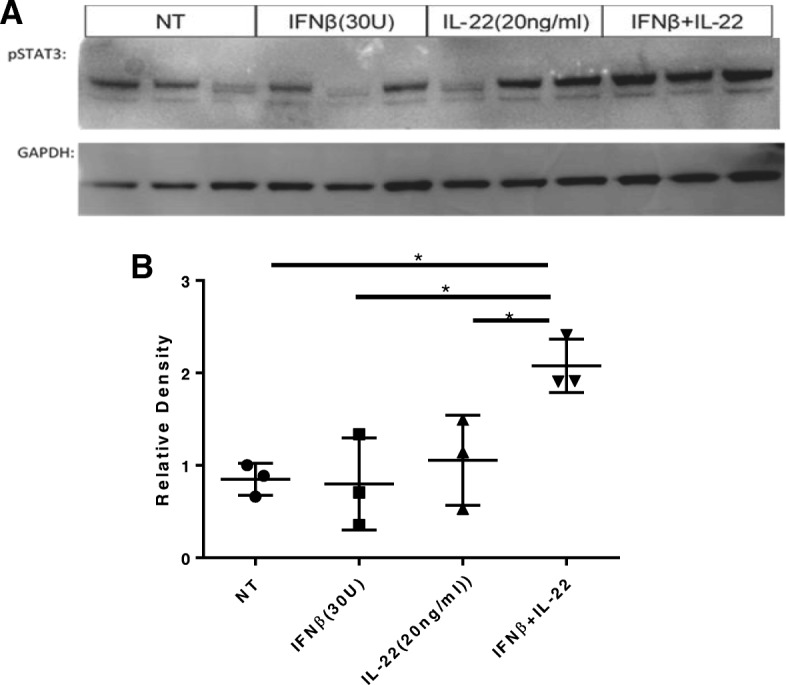
Enhanced IL-22 pathway activation after priming with IFNβ. BEAS-2Bs were pretreated with 30 U/ml of IFNβ for 24 h. Cells were then treated with human IL-22 (20 ng/ml) for 15 min. Cell were then collected in RIPA lysis buffer and protein isolated for **a** western blot analysis of pSTAT3 (Y705) normalized by GAPDH. **b** Densitometry analysis revealed that IFNβ pretreatment significantly enhances IL-22 signaling compared to IL-22 treatment alone (one-way ANOVA, *p* ≤ 0.0460). Representative of two experiments

To further investigate the regulation of IL-22Ra1, STAT inhibition assays were done to determine which STATs were indispensable to the induction of the receptor. We found that upon STAT1 inhibition, induction of *IL-22Ra1* was lost by both Poly (I:C) (one-way ANOVA, *p* = 0.0372) and IFNβ (one-way ANOVA, *p* ≤ 0.05) respectively (Fig. 5a and b). STAT3 inhibition did not affect induction of the receptor (data not shown). This was also confirmed in vivo as Poly (I:C) did not induce *Il22ra1* in STAT1^−/−^ mice (Fig. 5c). Taken together, these results demonstrate that the IL-22Ra1 is directly upregulated by type I interferon signaling via TLR3 activation by H1N1 in a STAT1 dependent manner (Figs. 6 and 7).

### Physiological relevance of IL-22Ra1 upregulation on epithelial cells

To test the relevance of IL-22Ra1 induction, cells were pretreated with IFNβ for 24 h prior to IL-22 administration. Western blot analysis of pSTAT3(Y705), the major IL-22 signaling pathway, revealed increased IL-22 signaling after pretreatment than IL-22 treatment alone (one-way ANOVA, *p* ≤ 0.05) (Fig. 7). Overall this illustrates that the rapid induction of IL-22Ra1 following H1N1 infection serves a physiological role to enhance IL-22 signaling.

## Discussion

Maintaining epithelial integrity in the lung is critical in fighting infection and allowing optimal gas exchange. Influenza viruses disrupt epithelial homeostasis as they directly target epithelial cells in the lung mucosa. Severe infection causes excessive epithelial injury, fluid buildup in the lung parenchyma and increased susceptibility to secondary bacterial infections [13]. Understanding how epithelial cells respond during influenza infection is important for optimizing immune based strategies to reduce the burden of the infection. Here we focused on the ability of epithelial cells to protect themselves through the induction of the IL-22 receptor. We demonstrated that the receptor is induced both in vitro and in vivo in a TLR3 dependent manner. This occurs in an IFNβ dependent manner as inhibition of the IFN receptor prevented *IL-22Ra1* induction. Moreover, using STAT inhibitors, we were able to demonstrate the *IL-22Ra1* induction involved STAT1 but not STAT3. Most importantly, the induction of IL-22Ra1 made the cells more responsive to IL-22 as seen by increased phosphorylation of STAT3.

IL-22 is beneficial at mucosal surfaces [14]. In the lung it is crucial for the production of anti-bacterial and anti-fungal agents [15, 16], and important for epithelial repair [10, 17]. These same proliferative and anti-apoptotic properties of IL-22 can also be detrimental as high levels are associated with, and also promote, hyperproliferation [18] and cancer [19, 20]. To prevent this from happening, IL-22 is tightly regulated. In the lung, IL-22 is not found under naïve conditions but is produced by NKT cells during the initial innate response (48–72 h) to influenza infection [17, 21]. Once produced, IL-22 initially acts upon cells in the upper and lower airways [15, 17, 21]. Production does not necessarily mean IL-22 will get to the target cells though, as the soluble inhibitor, IL-22 binding protein (IL-22BP), is constitutively produced in the lung, preventing IL-22 from binding to the IL-22Ra1 complex [22, 23]. This barrier can be overcome through downregulation of IL-22BP [24, 25] or optimization of IL-22 signaling at the target cell. Importantly, our data clearly demonstrate that increased receptor expression leads to increased IL-22 signaling and may be an important mechanism for optimizing IL-22 signaling in the IL-22/IL-22Ra1/IL-22BP axis.

IL-22Ra1 is highly conserved between mice and humans. At the nucleotide level it is 77.7% identical and at the amino acid level it is 71.9% identical [26]. Most importantly all three N-glycosylation sites which confer viability and functionality of the receptor are conserved as well as the STAT binding motifs [26]. The IL-22 receptor complex is a heterodimer of IL-22Ra1 and IL-10Rb. Here we demonstrate in both mouse and human models that influenza significantly induces IL-22Ra1 but not IL-10Rb (data not shown). These data are supported by in silico analysis of the GEO profiles data base in which *IL-22Ra1* was increased in H1N1 infection of human primary airway cells [27, 28]. This induction was stronger in pandemic infection compared seasonal infection and could also occur through IFNβ [28]. These data strongly corroborate our data and suggest that induction of the IL-22 receptor is important for epithelial cell survival during influenza infection.

Host cells have a number of pattern recognition receptors (PRRs) capable of recognizing infection. In the case of influenza, TLR3 and RIG-I recognize either double or single stranded RNA [4] and both have been implicated in optimizing the host cellular response to influenza [29]. TLR3 recognition of dsRNA requires intracellular vesicle formation in contrast to cytoplasmic RIG-I. In our experiments, the use of chloroquine, an endosomal inhibitor, completely prevented *IL-22Ra1* induction confirming TLR3 as the primary mediator in this pathway. TLR3 recognizes double stranded RNA (dsRNA) which is a common replication intermediate among all viruses [30]. Therefore, this pathway and IL-22 may be important in epithelial protection in other pulmonary viral infections in which the pulmonary epithelium is targeted. Interestingly, treatment with endotoxin (LPS), which is a bacterial byproduct that signals through TLR4, did not stimulate *IL-22Ra1* induction suggesting this may be a virus-specific phenomenon at the pulmonary mucosal surface.

To understand if *IL-22Ra1* was a primary or secondary TLR3 response gene [31] we targeted type I interferons as they are the most common TLR3 induced genes after influenza infection. Using an inhibitor of the type I interferon receptor, IFNAR2, we verified that TLR3 induction of *IL-22Ra1* required type I interferon. Interferons mediate clearance of viral pathogens via upregulation of hundreds of interferon-stimulated genes (ISGs) [8, 32]. ISG induction results in an antiviral state that functions to inhibit the virus at every stage of infection [4]. Here we demonstrate that type I interferon, IFNβ, and to a lesser extent type II interferon, IFNγ, but not type III IFN, induced *IL-22Ra1*.

Both type I and type II interferons signal through STAT pathways and share STAT1 as a signaling intermediate. In the case of Type I IFNs, STAT1 becomes phosphorylated and forms a heterodimer with activated STAT2 protein [32]. This is different for Type II interferon signaling in which STAT1 forms a homodimer upon activation. *IL-22Ra1* has both STAT1 and STAT1:STAT2 promoter sites upstream in its promoter and promoter flanking regions according to the Eukaryotic Promoter Database [33, 34]. Since IFNβ induced *IL-22Ra1* more strongly than IFNγ, we conclude that optimal IL-22 receptor induction requires STAT1:STAT2 heterodimer formation. Furthermore, neutralization of the IFNβ receptor resulted in complete loss of *IL-22Ra1* levels confirming IFNβ as the direct source of IL-22 receptor induction. More importantly, this IFNβ driven induction of IL-22Ra1 led to increased IL-22 signaling. Being that IL-22 is known to contribute to barrier maintenance and repair, this work may have implications for other viral infections in the lung that induce IFNβ.

Interferons have an interesting genetic association with the IL-22 axis. The genes encoding three major components of the IL-22 pathway are adjacent to members of type II and III interferon families respectively. *IL-22* is adjacent to *IFNγ* on human chromosome 12. *IL-22Ra2* is closest relative to *IFNγR1* on human chromosome 6 and mouse chromosome 10. *IL-22Ra1* is neighboring *IFNλR1* on chromosome 1 in humans and chromosome 4 in mice. The reason for the close genetic association of these families is not clear. However, given the reparative nature of IL-22 [10, 17, 21, 35] and the inflammatory nature of interferons [8], one could assume these two have a reciprocal relationship. The induction of interferons in response to infection is required for immune activation and with that comes epithelial damage. To counteract this, a pathway that is regenerative in nature must be activated at the same time to mitigate damage caused by both the infection and the immune system. Concurrent with immune activation, interferons, type I and II, are also priming the epithelial cell for repair following infection via the upregulation of the IL-22 receptor.

## Conclusions

IL-22 has been established in the lung as a cytokine that is essential for repair following influenza infection [10, 17, 21, 35]. The current study reveals that induction of its receptor, IL-22Ra1, following IAV infection allows for enhanced IL-22 signaling. Furthermore, we determined that this occurs in a TLR3/ IFNβ/STAT1 dependent manner. Together, these data demonstrate the IL-22/IL-22Ra1 axis as a possible therapeutic target for viral lung infections that activate TLR3 signaling. Given TLR3 recognizes viral RNA, this pathway may have implications in other viral infections that target the pulmonary epithelium. Overall, this study provides an important insight into the mechanisms involved in IL-22Ra1 induction and its physiological relevance following influenza infection.

## Data Availability

All data generated or analyzed during this study are included in this article.

## Abbreviations

BEBM: Bronchial Epithelial Cell Basal Medium
DMEM: Dulbecco’s modified Eagles medium
FBS: Fetal bovine serum
H1N1 PR8: Mouse adapted influenza A virus
IAV: Influenza A virus
IFN: interferon
IL-22: Interleukin 22
IL-22Ra1: Interleukin 22 receptor alpha 1
NHBE: Normal human bronchial epithelial cells
PAMP: Pathogen associated molecular patterns
PRR: Pattern recognition receptors
STAT: Signal transducer and activator of transcription
TLR: Toll-like receptors
